# Hygiene

**Published:** 1879-07

**Authors:** 


### HYGIENE

The idea that water will prevent the decomposition of fecal matter must have had its origin in the nursery room. There it is a common practice to use water to prevent bad odors. The truth is, as before explained, that by the use of water the gases are absorbed in the beginning till the water becomes saturated, so that if the fecal matter be diluted with water, and not allowed to remain too long in the room, the air will only be poisoned to a slight extent. However, after a time, when the water becomes saturated with the gases, the result is even worse, inasmuch as the water, by disolving a part of the feces, increases the active surface, and brings about, in a great degree, the putrefaction and action of the air upon the feces.

It is well known that captains of vessels are often compelled to take in impure drinking-water from the mouths of rivers. In the barrels, promoted by the rolling of the ship, the above-described process goes on, and the water, as well as the space in which the barrels are stowed, is poisoned to a dreadful degree.

A subterranean sewer is filled in its whole length with water charged with organic matter. Everywhere this fermentation goes on; the bubbles continually rising can be seen with the naked eye. Between the level of the fluid and the sewer the gas accumulates, which escapes through the air-holes in the sidewalks or any other opening, and in that way is inhaled by the people. There is absolutely no remedy to prevent the escape of those gases and the fungoid spores. The smallest crack in the walls would be sufficient to allow them to escape, and the most complicated closures have after some time, proved useless.

The effect of the water is purely mechanical, and is only expected to prevent the accumulation of fecal matter. Practical experience has proven that this is only imperfectly done, even when large quantities of water are at our disposal, and the repeated and continual failures in this regard are all attributed to the insufficiency of water.

It is not necessary to prove that there never will be a sufficient quantity of water to keep sewers clean. It is argued that what one millon of gallons cannot effect ten millions may; but no city has ever yet been able to obtain water enough for that purpose.

The danger of the exhaling gases cannot be disputed—they are simply poisonous. Many console themselves with the belief that these exhalations cannot be so dangerous, because their olfactories are not always able to distinguish them. Human beings have been compared to animals, step-motherly treated. Our organs of smell are not always very acute. One can live in a neighborhood where cholera or typhus are epidemic without distinguishing a difference in the inhaled air between that of a healthy neighborhood. The London *Medical Times*, which treats this question purely in a sanitary view, (March 23, 1861, p. 306,) gives this good advice in selecting residences or homesteads.

"Take no rooms in the neighborhood of a principal sewer, because it is an established fact that a greater mortality and more sickness exists on the line of great sewers than in any other place, and this is caused by the dangerous air escaping from the air-holes and other crevices." And again, (in February, 1861, p. 202:) "Do our readers know how the sewers are ventilated? Have you not had the misfortune to live near a grate, from which, on cool mornings a vapor arises which creates an intolerable stench? Now these grates are the mouths from which the obnoxious matter from decomposing feces eminate, to be wafted through the streets into our houses. Do not forget that these gases, in a concentrated form, produce instantaneous death. In a statistical table compiled by Chief Engineer Conrad, in Holland, it is shown that the mortality in one thousand inhabitants is thirty-five, and that twenty out of this number die of diseases which have their origin solely by inhaling impure air and the use of impure water."

The Chief Sanitary Inspector of Holland, Dr. Egeling, estimates that twenty thousand people die yearly in the Netherlands on account of the impurity of the air and water. In places where the water is pure and the air is not poisoned by the exhalations from excrements the mortality does not reach fifteen in every one thousand. These statistics prove that in a city of one hundred thousand inhabitants one thousand five hundred die yearly in consequence of the imperfectness of the sanitary system in the removal of excrements, &c., and besides the actual deaths, if we take into account the vast amount of disease which prevents men from following their ordinary occupations, and which, in a great measure, arise from the same influences, it will be clearly seen that it is the duty of all legislative bodies, having charge of the public health, to introduce as early as possible a change.

Medical Director, Dr. Stieber, of Berlin, in his work on the canalization of Berlin, (1866, p. 51,) says: "In juxtaposition to the natural removal of excrements to the country, which has been done instinctively from time immemorial, is the idea of sewerage in cities, which has grown to be a real mania, if we take into account that, with an expense of millions, with the greatest engineering talent employed, with the overcoming of the greatest technical objections, to build subterranean labyrinths, which possess no other merit than to remove the excrements from our eyes, and give them, in their progress through miles of sewers, the best opportunity, under the influence of water, by the finest distribution, to accelerate decomposition, to conduct the poisonous sewer-gases directly into our houses, to carry the water, saturated with foul particles and charged with gases, down the river, to diminish the capacity of production and vegetation, and to poison, in the course of one year, hundreds of our fellow-beings by the use of impure water."

In view of these facts we may be pardoned if we term the expenditure of millions of dollars every year for sewers a spendthrift measure, the loss of the manure a robbery, and the poisoning of the air and water an encroachment on the health of the people, not only for the present, but for the future.

A reference to the official report of Messrs. Haywood and Bazalgetter, English Royal Engineers, (see reports Metropolitan Board of Public Works, 1865–66, pp. 121–132,) will show the many different experiments made to improve the ventilation of th[illegible]ers, but all of them convinced the Parliamentary Commission that even by the best methods the result has never justified the enormous cost, and that no other way could be found than to allow the gases for the future to continue to escape from the middle of the streets, and that to burn the gases by means of high chimneys would take two hundred and fifty furnaces for the city of London alone, at the cost of two millions of dollars, and a yearly cost of half a million for fuel, exclusive of the cost of labor, &c.

To disinfect the sewers of a large city chemically would be a worse undertaking than emptying the ocean with buckets. If we were to inquire of the native African, living remote from any signs of civilization, as to what remedy he would suggest against the dangerous gases which are generated during the decomposition of the foces passing through the sewers, the answer of the son of nature would undoubtedly be: "Don't pour any more feces in." Not a very ingenious or philosophical argument, but, nevertheless, a very striking one. The removal of human excrements by means of sewerage does not fulfill its purpose—the whole system rests on an incredible narrowmindedness. The object is to carry off the refuse as quick as possible out of the reach of human habitations, but this is effected so imperfectly that, in many cases, it is removed from one house at the cost and detriment of others. In addition to this, let us look at the immense expense. To carry off the feces through pipes, three hundred times its quantity of water is necessary. Four thousand tons of human excrement consequently requires one million two hundred thousand tons of water, even under the present imperfect method. The present system of sewerage acts as a destructive agent in a two-fold manner—the water destroys the feces as manure, and the feces pollute the water; in other words, we pay fabulous sums of money for destroying the very article which, if properly treated, would be worth millions of dollars.

It is evident that fecal matter should not be allowed to run into the sewers in that way; it is entirely lost to agriculture, for which it is by nature designed. It has, therefore, become an imperative necessity that measures should be adopted by which this offensive matter can be collected without detriment to the public health, and applied directly for the purpose of agriculture, and no system is complete which does not fulfill these requirements. The present method of carting away the night-soil is most objectionable. The horrible smell during warm weather—nay, at all times—and the still more outrageous stench during the operation of emptying the receptacles in which it is kept, is most disgusting and loathsome to all those who come in contact with it. The very occupation of a scavenger is most revolting to the nature of any human being. The stench and disagreeable odor is entirely destroyed by the application of my patent mixture. Sinks or pits must be condemned. The ingredients kept in them for months, and even years, lose most of their value for manure.

*Parturiunt montes, nascitur—ridiculus mus.*

### Recapitulation.

First we have a complicated water-closet and water-conducting contrivance, the expenditure of millions of dollars for sewers, which gives us back a great portion of the obnoxious matter in their devious course, finding their way into our lungs, attended by known results; then millions of dollars for conducting immense quantities of water from a distance (in some instances) of many miles, or with the help of powerful engines to pump the water out of rivers, &c.; and, again, at the cost of enormous sums of money, to conduct in pipes for hundreds of miles a material too diluted to be of a practical use. And as the result of all this enormous outlay, we have typhus all the year round; cholera visits us yearly for a shorter or longer period, and small-pox is fearfully on the increase; when if this matter was properly understood and its importance fully realized, not only could this vast expenditure be be saved, but the health of the people improved, and the agricultural interests greatly benefitted.

There is certainly, since the world began, no parallel in which common sense and reason have been so much wanting as on this particular subject, and in time to come this will stand as a unicum in the history of human aberrations, and coming generations will laugh to scorn our folly.

